# Case of Croup

**Published:** 1808-03

**Authors:** 


### *To the Editors of the Medical and Physical Journal.*

Gentlemen,

A Child about 5 years old was brought to me for assistance; it was said to be ill of a sore throat. On inspecting the internal fauces, the tonsils and uvula were found covered seemingly by one continued slough, in the circumference of which, there was a distinct line of inflammation. From this appearance in the throat, I was led to examine the child's body, but found no efflorescence on the skin; there was evident pyrexia, but no remarkable expression of ailment in the countenance, and the respiration was unimpeded. A calomel purgative was all I now gave it. The next morning early I was called to visit it; it had now a frequent stridulous cough, and the characteristic and laboured breathing of croup—with that distrest aspect and restlessness, that attend this portentous disease. I bled it, vomited it, repeated the calomel, and blistered its throat. In the evening there was no alleviation, but an evident aggravation of symptoms; the breathing was more difficult and audible, and a most distressing and unceasing restlessness had commenced, up and down, to and fro, continually. The child seemed indeed in the "agony of suffocation." The calomel was again repeated, the pediluvium ordered, and the inhalation of the steams of vinegar; this produced an immediate increased fit of coughing, in in the act of which, a film of coagulable lymph was thrown from the mouth; the child became relieved in its breathing, and slept soundly for three hours; a change quite opposite to my expectation and expressed opinion. It continued with a cough and hoarseness upon it for several days, but from hence it recovered.

The substance that was thrown up, was kept for my inspection; it had been in water several hours when I first saw it, notwithstanding which it still kept its form and tenacity, and seemed indeed a perfect membrane. It was of an irregular shape, and was of too large dimensions to have come from below the glottis; it was separated no doubt from the larynx, and its expulsion I apprehend saved the patient.

I am far from considering this as a case of true or idiopathic croup, nor have I to flatter myself on any novelty of treatment. It is presumed, however, that a knowledge of such a simple fact as this even may have its use. A recollection of it might, at least, serve to keep up our hopes in similar occurrences, and might encourage us to protract the medicinal treatment; which in infant patients particularly, partly from our own oftentimes ill judging conviction of its inefficacy, and partly from the misgivings of parents, and the officiousness of friends, is not always carried, perhaps, to that extremity of result which it ought.

Since writing the above, an infant at the breast in the same family was similarly attacked, and died, not from febrile exhaustion, but from the disease extending to the trachea and producing suffocation. Is it from cases of this sort that the idea of croup being infectious first originated?

---

Fungus Hæmatodes.—I beg leave to call the attention of practitioners to this disease; not that I have a single fact to offer about it arising out of my own experience, but that I think two of your correspondents, Mr. Shearly and Mr. Weaver, have taken too simple a view of it. Happy indeed would it be for many a sufferer under this dreadful malady, if we could reduce it to so simple a construction; to a mere nucleus of blood, as Mr. Weaver seems to consider it, capable of being "rooted out and extracted by the finger." We might then well dispense with all that "severity of prognostic," which, from the observations of Mr. Hey, we should be induced to pronounce on such a case.

Mr. Weaver asks, "what affinity, or analogy, is there

 between between cancer, and this coagulated effusion of blood?" It never could be contended that there was the least affinity between them. Neither, we think, is there the most distant analogy, between Mr. Weaver's case, cured by the mere "enucleation" of a coagulum of blood, and those so interestingly described to us by Mr. Hey, under the appellation of fungus hæmatodes. And who, from sad and manifold experience, holds out no encouragement but from operation. "I have now," says this Gentleman, seen sixteen or seventeen cases of this disease, and perhaps many more, when I was not sufficiently aware of its nature, and have not been able to effect a cure in any instance, but by amputation of the limb, when the seat of the disease was in the extremities." We are not enabled to say, whether Mr. Hey ever tried arsenic as an escharotic in this disease, but he more than once observes, "that almost every kind of escharotic was tried, but in vain."

I beg leave to suggest, that in the works of the "original Monro," there are cases to be found, if not strictly the same, at least, bearing a strong affinity to those given us by Mr. Hey; and which were alike unfavourable in their termination. Mr. Hey gives it as one of the distinguishing marks of this disease, that, "the tumour in one part, will afford the sensation of a deep seated fluid, while another part feels hard and uneven." In one of Monro's cases, analogous, if we mistake not, to these, "The tumour became soft in some parts, with a fluctuation of a liquor, while hard tubercles were felt at other parts." And another time, we have this great surgeon candidly confessing that the touch deceived him. He imagined by the feel that there was matter pent up, and thought by an incision, to have set the disease at liberty; but no pus followed the lancet. "The next day," says he, "I brought two surgeons in my neighbourhood to visit my patient, and having taken off all the dressings except the pledget which covered the orifice, I desired them to feel the swelling and give me their opinion of it. They both affirmed their having felt the fluctuation of matter under their fingers. When the last pledgets were taken away, a fungus appeared at the orifice." The patient did not long survive. Upon dissection, "the skin of all the leg appeared sound, and the tunica cellulosa and muscles were all degenerated into that pappy substance which had appeared as a fungus, and I could not distinguish one muscle from another, though I was at considerable pains to dissect them. The periosteum was every where separated from both tibia and fibula."

la." (Vide Case of anomalous tumour of the Leg.) "Wherever," says Mr. Hey, as corresponding to the above, "the fungus comes into contact with the muscles, they lose their natural redness, and become brown. They lose also their fibrous appearance, and cannot in every part be distinguished from the adipose membrane."

A PRACTITIONER IN DERBYSHIRE.

*January* 20, 1808.

